# A Wonder Concerning Doctors

**Published:** 1871-04

**Authors:** Ben. H. Pratt


### A WONDER CONCERNING DOCTORS.

BEN. H. PRATT, A. M.

I wonder if people generally wonder as much as I wonder. Wonderers are said to be visionaries; but surely few are sufficiently visionary to loose their practicality. Hence, among so many men who seem supremely practical, there cannot be a very large visionary element; ergo, few wonder extensively. Are wonderers then visionists, and therefore impractical? Am I then to include myself among the latter? Well, what of it?

I don't see any very close relation between what I've just said and what I'm about to say, and yet I know I wonder a good bit. I don't know why I should wonder so much about doctors, but I do. Perhaps it's because I once had a slight attack of doctor-on-the-brain myself, and during my disorder struggled through much Anatomy, less Physiology, and little Materia Medica—practiced slinging doctors' slang a good deal, and mingled in Esculapian circles sufficiently for impractical purposes. I say that perhaps that's why I do wonder about doctors. Well, aren't they a wonder? Come now! Just look around. Take any locality you please, and ponder upon its doctors. Pick them out one by one. Analyze them.—Isn't it queer? Isn't it almost funny? Every place has its *gruff* old doctor, whose growl is proverbial—whose profanity is notorious—whose slovenly dress is an individuality—whose mare and sulky are an institution--whose name carries a fear with it—scares all the children—frightens all the women. His attainments are not of an intellectual sort. You don't see his diploma staring you in the face. You daren't ask him where he graduated. He don't care a flip for your universities, your schools, your 'pathies, your 'ologies. But somehow you respect as well as fear that gruff old doctor. By some mysterious process you are convinced that his medical skill is wondrous. You don't know why you believe in him, but you can't deny that you do. The whole neighborhood deems him infallible, and notwithstanding his rough look and manner, your serious cases are all referred to him. The well avoid, but the sick long for that burly form. It is only the poor invalid that knows this singular man. To such he is all sympathy and heart-felt devotion. With such he is all gentleness and kindness. No mother more tender in her administrations around the sick bed than this singularly constituted doctor. He can coo as no dove can, in the chamber above stairs. He can growl and rave as no beast can at anything wrong below stairs—at any defiance or neglect of his imperial orders. His word is law to those who wait upon his patients, and let any knowing old woman advise *him* to her peril. This is the *people's* doctor, mind you. He don't soil *many* velvet carpets, nor splatter much marble with *his* mixtures. Only when Crœsus gets very sick—why then, you know—well—it's a case of can't help it, you see.

Then there's our *model* doctor—slick and wise—careful in his appointments all—deliberate in his opinion—not so conversational as he is dictatorial, making rather than participating in public opinion—very booky—heavy on the schools of medicine of his particular guild—theoretical to the extent of infatuation—fearfully and oft-times fatally consistent. He treats a sick man as a wise architect would repair a ruin. He tears him down to a peg just above the dead point, and with plummet and level then begins to re-build, to re-create—how like to a God! What's more singular, he learns naught from failure. So long as he kills *secundem artem*, graves don't terrify nor coffins blanch him. He has wise authority for his plan of treatment. He will refer you to learned theses upholding every step of his scientific murder. He will convince your brain, though your bowels don't, won't, can't accept his sophistry. You know him. He's the First Family M. D., except in serious cases, where, as I before remarked, it's a little bit dangerous to call him.—Old Gruffy's the sheet anchor then, and as he's not thin-skinned, don't "decline to interfere" with brother Firsfam Lee, M. D.'s practice.

The dapper doctor is a wonder, too. He's a bachelor, you know, and "has a very fine practice." The ailings of the little ones—too much confectionery—shocking bad colds—headaches—ailments of mature and immature womanhood which don't quite come up to the standard of a "sickness"—these make our nice fellow quite busy, and pay pretty well, too—better than you and I think. Why! don't he buy the very nobbiest clothes and wear the nattiest boots and perch the shiniest beaver in town? And don't he escort some dear patient to church, and opera, and concert, and ball, and sociable every blessed night? How could he do so if his practice were not remunerative. He's not much of an opinionist. He don't think it quite polite to snub an elderly dame who, he inwardly confesses, knows more about medicine than he. He's *so* polite, and *so* considerate, and *so* genteel, and *so* nice, and has *such* an air, and is *so* popular. Why, of course we forgive him his innocence—his shallow materia medica, knowing he's a harmless necessity in every community.

Not sure about our *pompous* Doctor, however. He's glib enough, and proud enough, and vain enough, and handsome enough, and knows enough, to be a good doctor if he will, and a prominent, nay, shining member of society. But we're not sure of him. He's the biggest wonder because enigmas are always wonders. When you feel that a man may and can kiss or stab you with impunity, you are excusable for having some interest in knowing just which of these two operations he's about to perform upon you. Not that kissing is always so delightful, but that stabbing is generally so uncomfortable, especially when bunglingly done. I say I'm never sure of him. Not because he don't belong to the Church—nor to the Good Templers—nor this, that or t'other society that is supposed to sanctify men. Not that. But there's a certain indescribable way he has of talking with you—of looking at you—of listening to you. He seems more interested in his success, than in his patients, and yet I can't affirm either. If it were policy for a patient to die, I don't say that I'd ride by his office and call some less enigmatical M. D.; and yet I couldn't help mentally discussing the propriety of so doing. He's a regular practitioner and all that—don't commit the unpardonable professional sin of advertising (that is, openly in the papers)—is in good standing in the local Medical Society—but there's a something not imaginary about him—a heartlessness, so to speak—an inability to inspire such a confidence as seems almost indispensable between physician and patient—in fact, you don't like him and can't tell why. You don't know, but you *think* that he had something to do with that case about which so much mystery clings, and bids fair to continue clinging, notwithstanding the watchfulness of interested parties. When asked concerning him, you simply remark, "well, I don't know about him"—which is not a complimentary reply when the accent falls upon the "know." That he has many friends—enough money—a fair practice—these we all admit. I wish I could be as sure about some other matters appertaining to our pompous Doctor before leaving him.

The *born* Doctor is quite another wonder to me. How I *have* wondered over him. Does he have an eighth sense which we may term the diagnostic sense? I sometimes could swear to it. How otherwise could he, yet a boy, know just the right thing to do and the proper thing to give in an emergency? for accidents do happen to boys of which dear mammas and papas are never the wiser. How otherwise does he "take to" medicine as soon as he is out of his teens, and guided almost solely by his own impulse and instinct, achieve success unaccountably? There's nothing that makes a competitor *so* mad as to see a young fellow, new in physic, blundering along into an enviable reputation for medical skill. We call him a lucky dog that will some day fetch up against a tough case, or run his head into a mal-practice noose; but the case don't come along, and the noose don't transpire. That's what makes ordinary doctors mad. I don't wonder at *that;* it's the careless chap with the "luck" that makes the wonder grow. He don't profess much medical lore. You can stump him in the metaphysics of physic. You can give him a poser in doctor's latin and not half try. You can trip him up on medical technicalities. But, you can't deny his ability to cure the curable, and he loves to tackle one of those "given-ups" of other astute doctors. Wonder? who wouldn't? But these same born doctors don't dwell everywhere. You'il only meet two or three of them in the course of your life. I wouldn't worry about them, but I must wonder. If I was a doctor I wouldn't practice within—well, say a hundred miles of one such.

Last and least, I wonder much about traveling quacks. These perigrinating, peripatetic, bamboozling essences of humbuggery—these leeches—these double suckers of health and money. I don't wonder *how* they live (I can see through that)—but *why* they live, that puzzles me. I wonder why somebody don't kill 'em, first. Then I wonder why the glorious company of editors don't blast them newspaporily. Third, I wonder why there are so many fools left to patronize them, especially after being humbugged once or twice before. Fourth, I wonder how far impudence, effrontery, brass, cheek, will not go with the American public. By all the ties that bind me to my country, race, kindred and fellow citizens, I can't see why WE should be especially marked for our love of being humbugged. In fact, I wonder more and more, and the wonder grows with what it feeds on. But I suppose that while folks are folks and doctors are doctors, (and there's slight chance of either the one or the other changing spot or skin,) there will be just enough to wonder, just as I wonder, why some things are thus and other things are so.

